# MicroRNA-1 prevents high-fat diet-induced endothelial permeability in apoE knock-out mice

**DOI:** 10.1007/s11010-013-1606-x

**Published:** 2013-03-07

**Authors:** Hua Wang, Hua-Qing Zhu, Feng Wang, Qing Zhou, Shu-Yu Gui, Yuan Wang

**Affiliations:** 1 Laboratory of Molecular Biology and Department of Biochemistry, Anhui Medical University, Hefei, 230032 Anhui People’s Republic of China; 2 Department of Oncology, The Affiliated Provincial Hospital of Anhui Medical University, Hefei, Anhui People’s Republic of China; 3 Department of Respiratory Disease, The First Affiliated Hospital of Anhui Medical University, Hefei, Anhui People’s Republic of China; 4 Key Laboratory of Gene Research of Anhui Province, Hefei, Anhui People’s Republic of China

**Keywords:** MicroRNA-1, Permeability, Myosin light chain kinase

## Abstract

The development of atherosclerosis (AS) is a multifactorial process in which elevated plasma cholesterol levels play a central role. As a new class of players involved in AS, the regulation and function of microRNAs (miR) in response to AS remain poorly understood. This study analyzed the effects of miR-1 (antagomir and mimic) on endothelial permeability and myosin light chain kinase (MLCK) expression and activity in the artery wall of apoE knock-out mice after feeding them a high-cholesterol diet. Further, we tested to determine whether that effects are involved in ERK phosphorylation. Here, we show that a high-cholesterol diet induces a significant decrease of miR-1 expression. Histopathologic examination demonstrated that miR-1 antagomir enhances endothelial permeability induced by high cholesterol and miR-1 mimic attenuated endothelial barrier dysfunction. Consistent with endothelial permeability, Western blotting, qPCR, and γ-^32^P-ATP phosphate incorporation showed that MLCK expression and activity were further increased in miR-1 antagomir-treated mice and decreased in miR-1 mimic-treated mice compared with those of mice receiving control miR. Further mechanistic studies showed that high-cholesterol-induced extracellular signal regulated kinase (ERK) activation was enhanced by miR-1 antagomir and attenuated by miR-1 mimic. Collectively, those results indicate that miR-1 contributes to endothelial barrier function via mechanisms involving not only MLCK expression and activity but also ERK phosphorylation.

## Introduction

Atherosclerosis (AS) is a pathological multifactorial process, which very often starts with endothelial dysfunction and at later stages results in the deposition of cholesterol and other substances that progressively accumulate within arteries, finally leading to the formation of plaques [1]. High plasma cholesterol is associated with the development of AS. It has been demonstrated that cholesterol may increase the endothelial permeability [2, 3]. However, the available data on the structural aspects of endothelial permeability in the arteries are limited.

Endothelial dysfunctions are key events in the pathogenesis of AS. Myosin light chain kinase (MLCK) has been shown to contribute to vascular inflammation by altering endothelial barrier function. Several laboratories have demonstrated the central importance of MLCK in regulating the contractile state of the endothelial cells and in modulating endothelial cells’ barrier function [4–6]. MLCK, which transfers the γ-phosphate from ATP to myosin, is essential for endothelial cell contraction [7, 8]. In endothelial cells, actin-myosin contraction is mainly mediated by MLCK-catalyzed myosin light chain (MLC) phosphorylation. The phosphorylation condition of MLC affects the permeability of cultured endothelial cells and intact venular endothelium [9–11].

Recent studies have uncovered important and unexpected roles for a family of small regulatory RNA molecules, known as microRNAs (miR; miRNAs) in the regulation of diverse aspects of cardiovascular diseases. miRs are a class of small non-coding RNAs (20–24 nucleotides), which primarily bind to the 3′ untranslated region of target mRNA and negatively regulate gene expression at the post-transcriptional level [12]. miRs are involved in a wide range of pathophysiological cellular processes including development, differentiation, growth, metabolism, survival/death, and tumor formation [13–15]. Aberrant expression of miRs has been linked to a number of cardiovascular pathological conditions, including AS. As such, miRs have been suggested as novel therapeutic targets for cardiovascular diseases [16–18]. Previous studies have demonstrated that miR-1 is associated with cardiac hypertrophy and heart failure [19]. However, it remains to be determined whether miR-1 plays a role in AS.

It has been well established that diet-induced AS can cause an increase in the permeability of the arterial endothelium to lipoproteins [20]. However, whether miR-1 would affect endothelial permeability in high-cholesterol diet mice has not been investigated. In this study, we report that mice fed a high-cholesterol diet plus miR-1(antagomir and mimic) showed marked differences in endothelial permeability compared to mice fed a high-cholesterol diet, which may be associated with MLCK expression and ERK phosphorylation.

## Materials and methods

### Reagents

The following reagents were purchased: Sulfo-NHS-LC-Biotin from Pierce Chemical Co.(Rockford, IL, USA); anti-MLCK monoclonal antibody form Sigma Co.(Saint Louis, MI, USA); γ-^32^P-ATP from Yahui Biomedical Engineering Co. (Beijing, China); and miRNA oligonucleotide from QIAGEN Co. (Shanghai, China). All other chemicals used were of the purest commercially available grade. Calmodulin and myosin regulatory light chain were the gifts from Dr. Zhi at University of the Texas Southwestern Medical Center, USA.

### Animal experimental procedures

All experiments were approved by the Institutional Animal Care and Use Committee of Anhui Medical University. apoE^−/−^ mice (from the Institute of Basic Medical Sciences of Peking Union Medical College) were weaned at 4 weeks of age, at which point mice were either placed on standard diet (control) or a high-fat diet(standard diet plus 2 % cholesterol and 5 % lard oil) for 16 weeks. At 12 weeks, some of the control and high-fat mice were sacrificed for miR-1 level detection and some more of the control and high-fat diet mice randomized into 4 groups: control (standard diet), control miR oligonucleotide (high-fat diet), miR-1 antagomir oligonucleotide (high-fat diet), and miR-1 mimic oligonucleotide (high-fat diet). Mice received 2 subcutaneous injections of 10 mg/kg miR-1 antagomir, miR-1 mimic, or control miR in the first week, spaced 2 days apart, and weekly injections of 10 mg/kg miR-1 antagomir, mimic, or control miR thereafter for 4 weeks. At sacrifice, the mice were anesthetized with isoflurane and exsanguinated by cardiac puncture. The mice were perfused with PBS, followed by 10 % sucrose in PBS. A portion of the aorta was embedded in optimal cutting temperature compound (OCT) medium and frozen immediately, and the remaining aorta was snap frozen under liquid nitrogen and stored at −80 °C.

### MiR-1 expression assay

Total RNA was extracted from the aorta of different groups using Total RNA Isolation Reagent (TRIzol reagent, Invitrogen). MiR-1 expression was determined using the miRNA plate assay kit (Signosis, Inc.,) according to the manufacturer’s instructions. For normalized RNA content, the U6 snRNA was the internal control.

### Permeability assay

The permeability assay of using the surface biotinylation technique was performed as described by Zhu et al. [20] with some modifications for the aorta intima. The aorta canals were filled with freshly made 1 mg/ml Sulfo-NHS-LC-Biotin in HBS (HBS containing 1 mM CaCl_2_ and 1 mM MgCl_2_) for 30 min at room temperature. The aortas were rinsed with PBS and embedded in OCT and cryosectioned. 10-μm frozen sections were incubated in blocking buffer (1 % fish skin gelatin and 1 % BSA in PBS) for at least 5 h. After washing three times with blocking buffer, sections were then incubated with X-Rhodamine Isothiocyanate (XRITC) avidin and examined by fluorescence microscopy.

### MLCK mRNA assay

Real-time PCR was used—Total RNA was extracted from the aorta of different groups using the TRIzol reagent (Invitrogen) following the manufacturer’s instructions. Real-time PCR for mice MLCK and glyceraldehyde-3-phosphate dehydrogenase (GAPDH) mRNA was performed. The primers for MLCK and GAPDH were described previously (10).

### Western blot analysis

The aortas were washed three times in PBS and then lysed in RIPA buffer (1 % Nonidet P-40, 1 % sodium deoxycholate, 0.1 % SDS, 150 mM NaCl, 10 mM sodium phosphate buffer pH 7.2, 2 mM EDTA, 10 mg/ml aprotinin, 10 mg/ml leupeptin, 2 mM PMSF, 2 mM sodium orthovanadate, 10 mM sodium pyrophosphate, and 20 mM sodium fluoride). The lysates were centrifuged at 15,000×*g* for 30 min at 4 °C. The total protein concentration of each sample was measured using the MicroBCA Protein Assay Reagent Kit (Pierce, Rockford, IL, USA). The same amount of lysate from each line in SDS sample buffer was electrophoresed with 10 % SDS-polyacrylamide gel and electroblotted onto a PVDF membrane, which was then blocked with 5 % fat-free milk in PBST (PBS, 0·1 % Tween 20) for 1 h at room temperature. Monoclonal antibody (1:1,000 dilution) was incubated overnight at 4 °C, followed by incubation with 1:2000 diluted HRP-conjugated goat antibody against rabbit IgG, and stained with enhanced chemiluminescence reagent (Pierce, Rockford, IL, USA). Densitometric scanning of the exposed X-ray film was used for semi-quantitative measurement of the protein bands. Three independent experiments were performed and the results were reproducible.

### Assay of MLCK activity

The activity of MLCK was measured by rates of γ-^32^P-ATP incorporation into MLC. The maximal activity was determined in the reaction buffer containing 50 mM MOPS, 10 mM magnesium acetate, 1 mM DTT, 0.3 mM CaCl_2_, 1 M γ-^32^P-ATP (200–300 cpm/pmol), 1.2 μM CaM, 25 μmol/l myosin regulatory light chain, and diluted MLCK at room temperature. MLCK was freshly diluted in 10 mM MOPS, 1 mM DTT, and 0.1 % BSA, added to the reaction mixture, and samples were incubated for 10 min at 30 °C. The reaction was terminated by filtering through Whatman paper. Filters were added to scintillation fluid and placed in a scintillation counter. Blanks were samples run without substrate.

### Statistical analysis

The data are expressed as the mean ± SD. A comparison among each group was performed by one-way analysis of variance followed by the Neuman–Keuls test to evaluate the statistical significance between two groups. *P* value of <0.05 was considered to be statistically significant.

## Results

### MiR-1 treatment influences miR-1 expression in the aorta

**Fig. 1:**
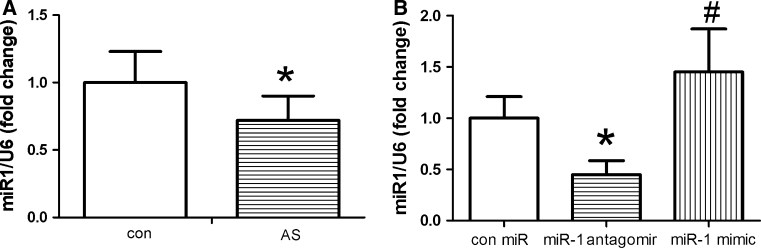
Total RNA was extracted from the aorta of different groups using TRIzol reagent; MiR-1 expression was determined using the miRNA plate assay kit; for normalized RNA content, the U6 snRNA was the internal control. **a** miR-1 expression was detected in control and AS mice. **b** miR-1 treatment influences miR-1 expression in the aorta. Levels of miR-1 were detected in different miR-treated AS mice, *P < 0.05 significance relative to Con, ^#^ *P* < 0.05 significance relative to miR-1 antagomir

To assess the effects of miR-1 in a model of established AS, apoE^−/−^ mice were first fed a high-cholesterol diet for 12 weeks, then injected subcutaneously with 10 mg/kg of miR-1 antagomir, mimic, or control miR oligonucleotides. To maximize miR delivery, mice were injected twice during the first week, then once weekly thereafter, for a total of 4 weeks. First, we measured the expression of miR-1 in the aorta of the mice. Levels of miR-1 detected by quantitative PCR were decreased in the high-cholesterol diet-fed mice compared with those of the standard diet-fed mice (see Fig. 1a). Further, to determine the efficacy of miR-1 treatment, we measured the expression of miR-1 in the aorta of mice after 4 weeks of treatment. Levels of miR-1 were decreased by more than 50 % in miR-1 antagomir-treated mice compared with those of mice receiving control miR. Consistent with this, the expression of miR-1 in the aorta was increased in miR-1 mimic-treated mice (see Fig. 1b).

### MiR-1 treatment influences endothelial permeability in the aorta

**Fig. 2:**
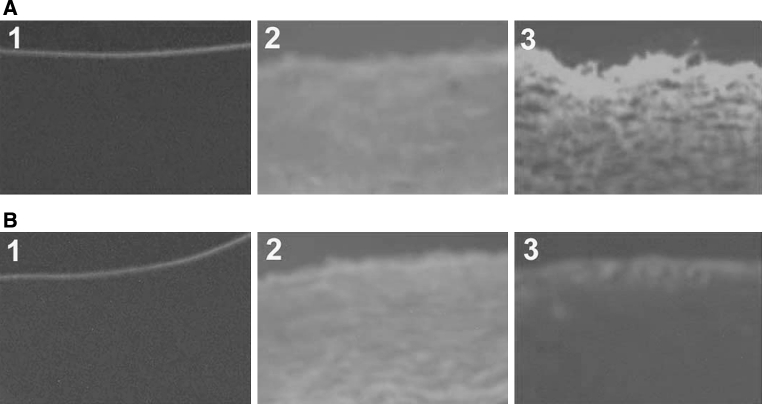
miR-1 treatment influences endothelial permeability in the aorta. After aorta intimas were incubated with NHSLC-biotin for 30 min, frozen sections were incubated with XRITC-avidin to localize surface-bound biotin. Photographs taken at 20-fold magnification. **a** 1: Con + Con miR; 2: AS + Con miR; 3: AS + miR-1 antagomir; B: 1: Con + Con miR; 2: AS + Con miR; 3: AS + miR-1 mimic; *P < 0.05 significance relative to Con, ^#^ *P* < 0.05 significance relative to AS + Con miR

To demonstrate the effect of miR-1 on endothelial permeability, the transport of NHSLC-biotin across the aortic intima to the media was determined. Concentration profiles of NHSLC-biotin were obtained as a function of the radial distance through the media of the aortic wall. Only the endothelium surface of the aorta intima was biotinylated in the normal diet mice, indicating no paracellular leakage of NHS-LC-biotin (Fig. 2a1, b1). The aorta intimas in AS mice were incubated with NHS-LC-biotin (Fig. 2a2, b2). The leakage of NHS-LC-biotin into the aorta intima further increased in miR-1 antagomir-treated mice (Fig. 2a3), compared with those of mice receiving control miR (Fig. 2a1); consistent with this, endothelial permeability in the aorta was attenuated in miR-1 mimic-treated mice (Fig. 2b3).

### MiR-1 treatment influences MLCK expression and activity in the aorta

**Fig. 3:**
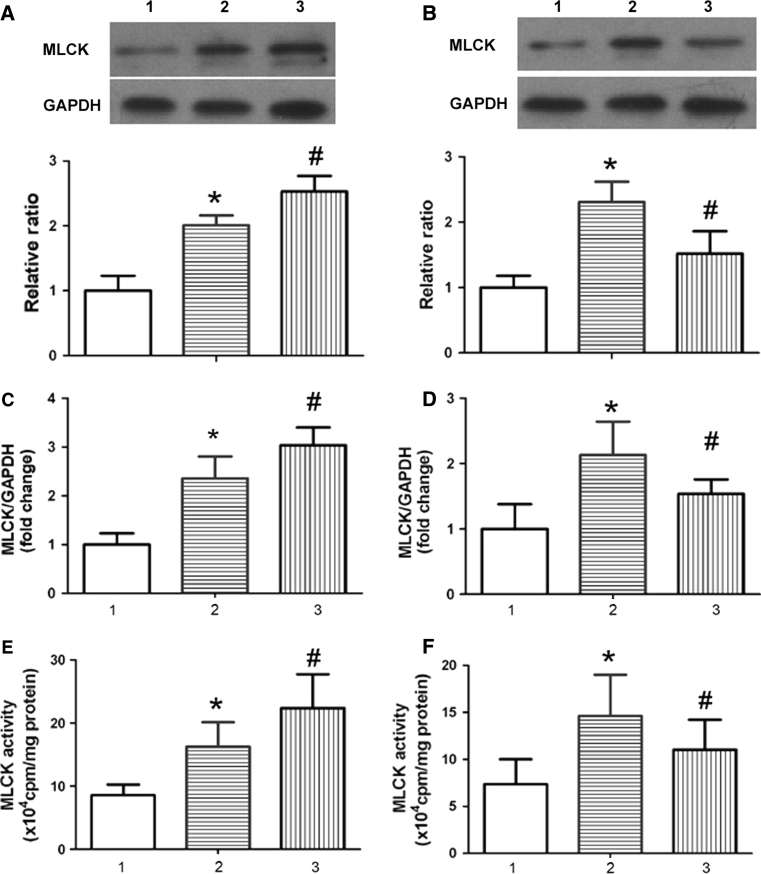
miR-1 treatment influences MLCK expression and activity in the aorta. Western blotting (**a**, **b**) and qPCR (**c**, **d**) were performed as described in the “Materials and methods” section to detect MLCK expression, and γ-^32^P-ATP phosphate incorporation was performed to assay MLCK activity(E and F). A,C,E: 1:Con + Con miR; 2: AS + Con miR; 3: AS + miR-1 antagomir; B,D,F: 1:Con + Con miR; 2: AS + Con miR; 3: AS + miR-1 mimic; *P < 0.05 significance relative to Con, ^#^ *P* < 0.05 significance relative to AS + Con miR

With the knowledge that endothelial barrier function is maintained by a delicate balance between tethering and contractile forces that involve the cytoskeleton, we sought to determine whether MLCK expression and activity are involved in AS mice. Western blot and qPCR were performed and showed that expression of MLCK in the AS aorta was higher than that of control in the aorta intima. MLCK expression was further increased in miR-1 antagomir-treated mice compared with those of mice receiving control miR. Consistent with this, the expression of MLCK in the aorta was decreased in miR-1 mimic-treated mice (Fig. 3a–d). Further studies demonstrated that MLCK activity is also necessary for high-cholesterol-induced AS. The results indicated that the MLCK activity in the aorta was enhanced in miR-1 antagomir-treated mice and reduced in miR-1 mimic-treated compared with those of mice receiving control miR (Fig. 3e, f).

### MiR-1 treatment influences ERK phosphorylation in the aorta

**Fig. 4:**
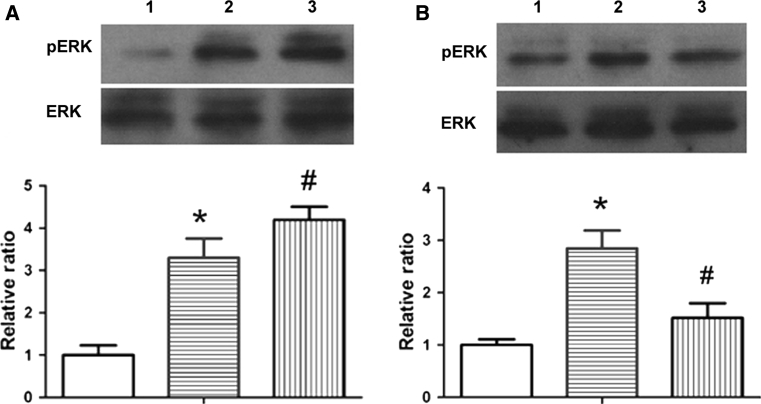
miR-1 treatment influences ERK phosphorylation in the aorta. Control and AS mice were injected with different miRs. Four weeks later, phosphorylated ERK (pERK) and total ERK (ERK) were determined in aortic tissues by western blot analysis. The upper panel is a representative western blot for pERK and ERK and the lower panel is the quantitative data from western blot analysis for the ratio of pERK/ERK. **a** 1: Con + Con miR; 2: AS + Con miR; 3: AS + miR-1 antagomir; **b** 1: Con + Con miR; 2: AS + Con miR; 3: AS + miR-1 mimic; *P < 0.05 significance relative to Con, ^#^ *P* < 0.05 significance relative to AS + Con miR

To explore whether high-cholesterol-induced ERK activation is involved in MLCK expression and activities, phosphorylation of ERK was measured in AS mice. As shown in Fig. 4, high-cholesterol-induced ERK activation was enhanced by miR-1 antagomir and attenuated by miR-1 mimic; similar results of MLCK expression and activity were observed in high-cholesterol-induced mice, indicating that high-cholesterol-induced MLCK expression and activity were associated with the phosphorylation of ERK.

## Discussion

The novelty of our study is that miR-1 contributed to endothelial cell permeability, which was associated with regulation of MLCK expression via mechanisms involving ERK phosphorylation. Vascular endothelial cells serve as cellular barriers to the circulating blood. There have been many ultrastructural studies on arterial endothelial damage in animals fed a high-cholesterol diet [2, 20], but few have referred to the relationship between AS and miR-1 and its associated permeability.

In the present study, AS was caused in the mice using a cholesterol-rich diet. It has been demonstrated that high plasma cholesterol is associated with the development of AS as previously reported [2, 21, 22]. It is shown that the permeability of the arterial wall and the expression of MLCK increased after the mice were fed cholesterol for 12 weeks. MiR-1 mimic attenuated the permeability and MLCK expression. The results also indicated that the MLCK activity in the aorta was enhanced in miR-1 antagomir-treated mice and reduced in miR-1 mimic-treated compared with those of mice receiving control miR. From those results, it is hypothesized that the change of integrity of the arterial wall may be associated with the miR-1 and MLCK expression and activity.

The functions of various miRs and their involvement in biologic processes have been identified in animal models or various cultured cells. The expression profiles of circulating miRs with cardiovascular diseases have been extensively studied, and it was found that miR-21, -34a, -146a, -146b-5p, and -210 were expressed at significant levels in AS [23, 24].Unfortunately, whether miR-1 would affect endothelial permeability in high-cholesterol diet mice has not been investigated and the involvement of miR-1 in high-cholesterol diet mice has received little attention. Our studies showed that MLCK expression and activity were further increased in miR-1 antagomir-treated mice and decreased in miR-1 mimic-treated mice compared with those of mice receiving control miR.

ERK/Mitogen-activated protein kinase (MAPK) pathway is a key signal transduction pathway and is associated with many inflammatory diseases [25]. This pathway is activated by many cytokines and growth factors which are produced under a state of stress and injury. Previous studies have indicated that cell adhesion is mainly dependent on ERK [26, 27]. Therefore, the development of AS may be associated with the ERK/MAPK pathway. When ERK/MAPK is activated, these proteins rapidly translocate to the nucleus where they combine with target genes and upregulate their expression. In endothelial cells damaged by cholesterol, MAPK protein will overexpress cell adhesion molecules through the ERK/MAPK pathway [28]. This study indicated that cholesterol significantly increases phosphorylation of ERK in vascular endothelial cells. This study showed that high-cholesterol-induced ERK activation was enhanced by miR-1 antagomir and attenuated by miR-1 mimic.

In summary, the present study provides experimental evidence supporting the contribution of the endothelial cytoskeleton to the pathological regulation of endothelial barrier function. MiR-1 and MLCK were suggested to play important roles in the development of atherosclerosis. However, the exact mechanism to induce MLCK expression was not known. This would be followed by a series of events including activation or expression of a protein phosphatase, phosphorylation or dephosphorylation of a protein, activation or inhibition of its activity, and finally affecting gene transcription. In a conclusion, MLCK activity and expression influenced by miR-1 may be involved in many signal transductions, which remain to be elucidated in our future study.

## References

[1] Thum T (2012). MicroRNA therapeutics in cardiovascular medicine. EMBO Mol Med.

[2] Libby P, Ridker PM, Hansson GK (2011). Progress and challenges in translating the biology of atherosclerosis. Nature.

[3] Collins NT, Cummins PM, Colgan OC (2006). Cyclic strain-mediated regulation of vascular endothelial occludin and ZO-1: influence on intercellular tight junction assembly and function. Arterioscler Thromb Vasc Biol.

[4] Huang Q, Xu W, Ustinova E (2003). Myosin light chain kinase-dependent microvascular hyperpermeability in thermal injury. Shock.

[5] Stroka KM, Aranda-Espinoza H (2011). Endothelial cell substrate stiffness influences neutrophil transmigration via myosin light chain kinase-dependent cell contraction. Blood.

[6] Hicks K, O’Neil RG, Dubinsky WS (2010). TRPC-mediated actin-myosin contraction is critical for BBB disruption following hypoxic stress. Am J Physiol Cell Physiol.

[7] Isotani E, Zhi G, Lau KS (2004). Real-time evaluation of myosin light chain kinase activation in smooth muscle tissues from a transgenic calmodulin-biosensor mouse. Proc Natl Acad Sci USA.

[8] Hata T, Goto C, Soga J (2011). Measurement of Rho-associated kinase (ROCK) activity in humans: validity of leukocyte p-MBS/t-MBS in comparison with vascular response to fasudil. Atherosclerosis.

[9] Kaur S, Leszczynska K, Abraham S (2011). RhoJ/TCL regulates endothelial motility and tube formation and modulates actomyosin contractility and focal adhesion numbers. Arterioscler Thromb Vasc Biol.

[10] Reynoso R, Perrin RM, Breslin JW (2007). A role for long chain myosin light chain kinase (MLCK-210) in microvascular hyperpermeability during severe burns. Shock.

[11] Tinsley JH, Teasdale NR, Yuan SY (2004). Myosin light chain phosphorylation and pulmonary endothelial cell hyperpermeability in burns. Am J Physiol Lung Cell Mol Physiol.

[12] Tan G, Shi Y, Wu ZH (2012). MicroRNA-22 promotes cell survival upon UV radiation by repressing PTEN. See 1 citation found by title matching your search. Biochem Biophys Res Commun.

[13] Bushati N, Cohen SM (2007). MicroRNA functions. Annu Rev Cell Dev Biol.

[14] Chang TC, Mendell JT (2007). MicroRNAs in vertebrate physiology and human disease. Annu Rev Genomics Hum Genet.

[15] Duan X, Ji B, Wang X, Liu J (2012). Expression of microRNA-1 and microRNA-21 in different protocols of ischemic conditioning in an isolated rat heart model. Cardiology.

[16] Weber C, Schober A, Zernecke A (2012). MicroRNAs in arterial remodelling, inflammation and atherosclerosis. Curr Drug Targets.

[17] Nazari-Jahantigh M, Wei Y, Schober A (2012). The role of microRNAs in arterial remodelling. Thromb Haemost.

[18] Qin S, Zhang C (2011). MicroRNAs in vascular disease. J Cardiovasc Pharmacol.

[19] Elia L, Contu R, Quintavalle M (2009). Reciprocal regulation of microRNA-1 and insulin-like growth factor-1 signal transduction cascade in cardiac and skeletal muscle in physiological and pathological conditions. Circulation.

[20] Zhu HQ, Zhou Q, Jiang ZK (2011). Association of aorta intima permeability with myosin light chain kinase expression in hypercholesterolemic rabbits. Mol Cell Biochem.

[21] Liu HR, Tao L, Gao E (2004). Anti-apoptotic effects of rosiglitazone in hypercholesterolemic rabbits subjected to myocardial ischemia and reperfusion. Cardiovasc Res.

[22] Birukova AA, Birukov KG, Adyshev D (2005). Involvement of microtubules and Rho pathway in TGF-beta1-induced lung vascular barrier dysfunction. J Cell Physiol.

[23] Chen LJ, Lim SH, Yeh YT (2012). Roles of microRNAs in atherosclerosis and restenosis. J Biomed Sci.

[24] Nazari-Jahantigh M, Wei Y, Noels H (2012). MicroRNA-155 promotes atherosclerosis by repressing Bcl6 in macrophages. J Clin Invest.

[25] Zhu HQ, Cheng XW, Xiao LL (2008). Melatonin prevents oxidized low-density lipoprotein-induced increase of myosin light chain kinase activation and expression in HUVEC through ERK/MAPK signal transduction. J Pineal Res.

[26] Wang J, Zhou JY, Wu GS (2007). ERK-dependent MKP-1-mediated cis-platin resistance in human ovarian cancer cells. Cancer Res.

[27] Zhou JY, Liu YS, Wu GS (2006). The role of mitogen-activated protein kinase phosphatase-1 in oxidative damage-induced cell death. Cancer Res.

[28] Wang YQ, Dai M, Zhong JC (2012). Paeonol inhibits oxidized low density lipoprotein-induced monocyte adhesion to vascular endothelial cells by inhibiting the mitogen activated protein kinase pathway. Biol Pharm Bull.

